# Supplementary material for: Risks posed by invasive species to the provision of ecosystem services in Europe
Source: Nat Commun. 2024 Apr 10;15:2631. doi: 10.1038/s41467-024-46818-3 (PMC11006939; doi:10.1038/s41467-024-46818-3)
Supplement: Supplementary file 1 — Supplementary Information [file 41467_2024_46818_MOESM1_ESM.pdf]

Supporting information for:

Risks posed by invasive species to the provision of ecosystem services in Europe

Gallardo B., S. Bacher, A. M. Barbosa, L. Gallien, P. González-Moreno, V. Martínez-Bolea, C. Sorte, G. Vimercati and M. Vilà

Corresponding author: Belinda Gallardo (belinda@ipe.csic.es)

This PDF file includes:

Supplementary Methods

Supplementary Figures 1 to 12

Supplementary Tables 1 to 2

Supplementary References

Supplementary Methods

Sensitivity Analysis: Current Exposure

The calculation of current exposure depends on the thresholds used to classify sites into Low, Medium and High delivering areas. We therefore performed sensitivity analysis using three alternative breakdowns of ecosystem services values:

- *Alternative 1*: Low (ES ≲10%), Medium (10-90% ES) and High (ES ≳90%)
- *Alternative 2* (selected for this study): Low (ES ≲20%), Medium (20-80% ES) and High (ES ≳80%)
- *Alternative 3*: Low (ES ≲30%), Medium (30-70% ES) and High (ES ≳70%)

Patterns of exposure to invasive species upon the 7 ES were consistent along the three alternative thresholds investigated, with the average number of invasive species present per category showing a variation of 5.8% (between Alternative 2 and 3) and 6.6% (between Alternative 1 and 2) (Supplementary Fig. 5). Differences in current exposure among sites providing Low, Medium and High ES were significant in the three alternatives (Kruskal-Wallis Test, $P<0.001$ in all cases). This is probably caused by the large sample size of analysis covering the whole of Europe (N between 2709 and 21318 depending on the NCP considered, see Supplementary Table 4). The current exposure was significantly lower in High provisioning areas for Habitat maintenance, soil retention, flood control and outdoor recreation. The contrary was observed for N retention, the provisioning of crops and timber, in which case there was a higher exposure to invasive species in High delivering areas.

Modelling invasive species potential distribution

To ensure that our choice of modelling method did not bias our results and conclusions, we compared BART predictions to those obtained with three other methods of different complexity, all of which have previously been shown to provide robust results in species distribution modelling: Generalised Linear Models (GLM), computed with the 'glm' function of base R; Generalised Additive Models (GAM), computed with the 'gam' function of the 'gam' R package v 1.22-2; and Generalised Boosted Models (GBM, also known as boosted regression trees), computed with the 'gbm' function of the 'gbm' R package v 2.1.9.

Models were subjected to five-fold spatial block cross-validation. Unlike conventional random cross-validation, which may underestimate prediction error on spatially structured or clustered data samples [1], block cross-validation spatially separates the training and testing data, allowing a more rigorous assessment of model predictive ability, in both nearby and more distant locations [2]. The study area (i.e. the modelling buffer) of each species was divided into five folds (groups) of spatial blocks, using the ‘spatialBlock’ v 2.1.4 function of the ‘blockCV’ R package v 2.1.1 [2]. The size of these spatial blocks was set to 200 km, which was considered a reasonable distance beyond which similar presence and prediction patterns are no longer simply due to spatial autocorrelation. Larger block sizes, defined e.g. by the range of spatial autocorrelation in predictor variables [2], reduce the environmental representation in model testing blocks, thereby producing unreasonably low evaluation scores [1]. We modelled the distribution of each species five times, each time leaving out one of the five folds of spatial blocks. Each of these five partial models was then tested on the corresponding reserved fold. Finally, the mean and variance of

the model evaluation metrics were assessed across folds. After assessing this cross-validation performance, a final model was computed for each species using the entire dataset.

Results from cross-validation indicated good to excellent performance of distribution models, with an average AUC of 0.86 ± 0.06 and MCS of 1.09 ± 0.18. *Limnoperna fortunei* was the only species achieving AUC < 0.7, which can be considered poor. Two species obtained MCS slightly above 1.5 (i.e. far from 1). Cross-validation performance indicators (AUC and MCS) for the 94 Invasive species investigated across the 4 algorithms (BART, GLM, GAM and GBM) can be found in Supplementary Table 5. Considering the high inter-correlation among candidate variables shown in Supplementary Fig. 6, identifying the most important predictors of individual invasive species requires more detailed investigation that is beyond the scope of this study. Accessibility, bio6 (temperature of coldest month), bio 13 (precipitation of wettest month), bio 4 (temperature seasonality), and bio 12 (annual precipitation) were among the most important predictors of the distribution of our focus invasive species, with slight changes across major taxonomic groups (Supplementary Fig. 7). Temperature related predictors (bio 1 to bio 11) contributed 54 ± 6% to the models; whereas the overall contribution of precipitation related predictors (bio 12 to bio 19) was lower, at 36 ± 5%.

Sensitivity analysis: Range expansion

Potential range expansion, measured as total number of additional 10x10 km cells that are favourable for the establishment of invasive species, can be sensible to the threshold used to turn continuous maps of favourability (0-100%) into binary maps of likely presence/absence of species (0/1). We therefore investigated range expansion using three alternative thresholds:

- *Alternative 1*: favourability ⋝ 90%
- *Alternative 2*: favourability ⋝ 80%
- *Alternative 3*: favourability ⋝ 70%

The total area classified as suitable for invasive species increased from Alternative 1 to Alternative 3. In comparison with the total area currently occupied by invasive species (corresponding to "Current threat" in Fig. 8), Alternative 1 results in an average range contraction of 3% for invasive species of European concern, which suggests that this option is too conservative when looking at the areas most suitable for future invasion. In contrast, Alternative 3 results in a range expansion of 163% on average across the 94 invasive species investigated. Under this alternative, the area occupied by invasive species in Europe almost triples, which is possible but unrealistic in the near future. At an intermediate level, Alternative 2 results in a range expansion of 77% relative to the area currently invaded. This is considered the most plausible option and is therefore used in the main manuscript (Fig. 3).

Some invasive species showed a very high potential for range expansion, particularly when they currently occupy a minimal fraction of their potential niche (e.g. *C. candensis, L. getula, S. nige*r, Fig. 3). At the other extreme we find invasive species with minimal potential for range expansion; this was the case of species that seem to have filled most of their climate niches (e.g. *O. jamaicensis, A. syriaca*) or that may even lose suitable habitat (e.g. *A. triangulatus, L. catesbeianus*).

Sensitivity analysis: Risk posed by invasive species to ecosystem services

We compared the total area of Europe classified into each of the 9 potential risk categories using three alternative ecosystem services (ES) thresholds:

- *Alternative 1*: Low (ES ⋜10%), Medium (10-90% ES) and High (ES ⋝90%)
- *Alternative 2* (selected for this study): Low (ES ⋜20%), Medium (20-80% ES) and High (ES ⋝80%)
- *Alternative 3*: Low (ES ⋜30%), Medium (30-70% ES) and High (ES ⋝70%)

Patterns were consistent across the three alternatives, although the area of Europe classified in the extreme categories (Low and High) arguably increased from Alternative 1 to 3, simply because a larger percentile of data is considered (Supplementary Fig. 10).

Supplementary Figures

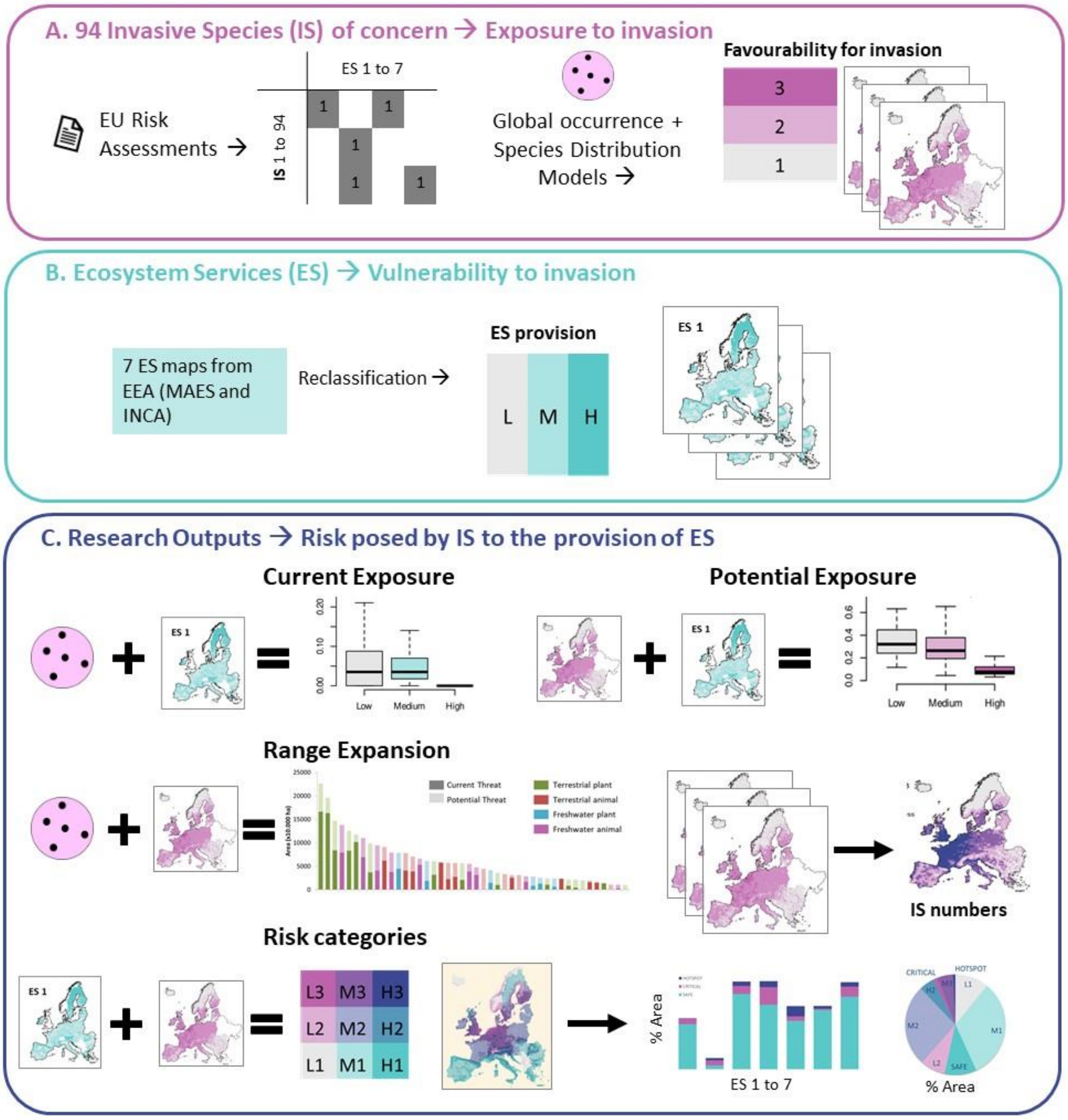

Supplementary Fig. 1. *Workflow of the analyses implemented to investigate the risks posed by invasive species to the provision of ecosystem services. A) We compiled risk assessments and the global occurrence of 94 invasive species of concern in Europe, including animals and plants from terrestrial and aquatic environments. We used Species Distribution Models to anticipate their potential distribution and reclassified maps into 3 categories of favourability for invasion. B) We compiled maps of the provision of 7 regulating, provisioning and cultural services in Europe and reclassified them into 3 categories of provision. C) We combined data to calculate: the current exposure, the potential exposure, and range expansion. Finally, we combined favourability for invasion and ecosystem service provision to investigate the total area and spatial distribution of 9 categories of risk.*

Supplementary Fig. 2. *Occurrence of 94 invasive species of European concern. This information was used to calibrate species distribution models. Sources of information can be consulted in Supplementary Table 2.*

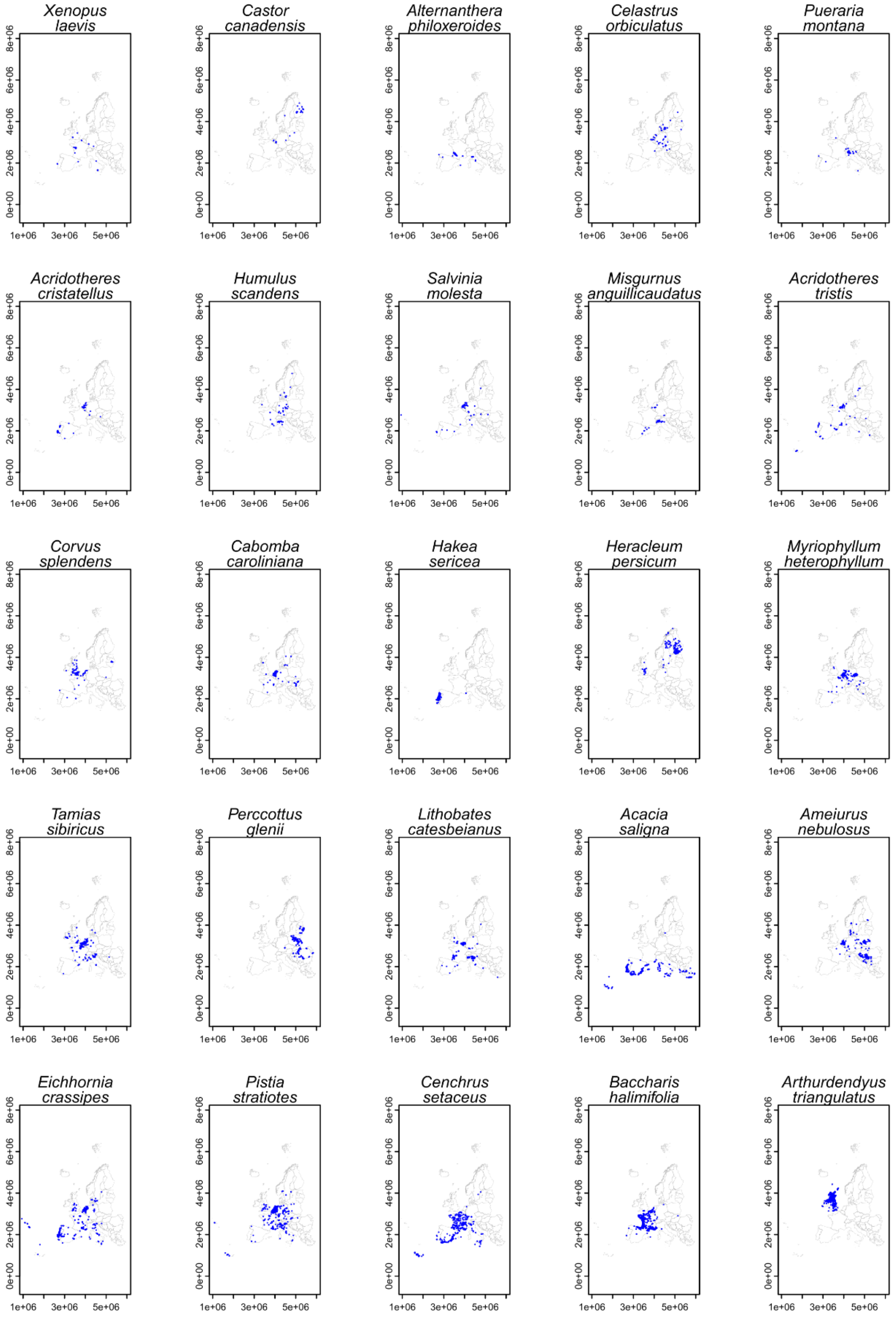

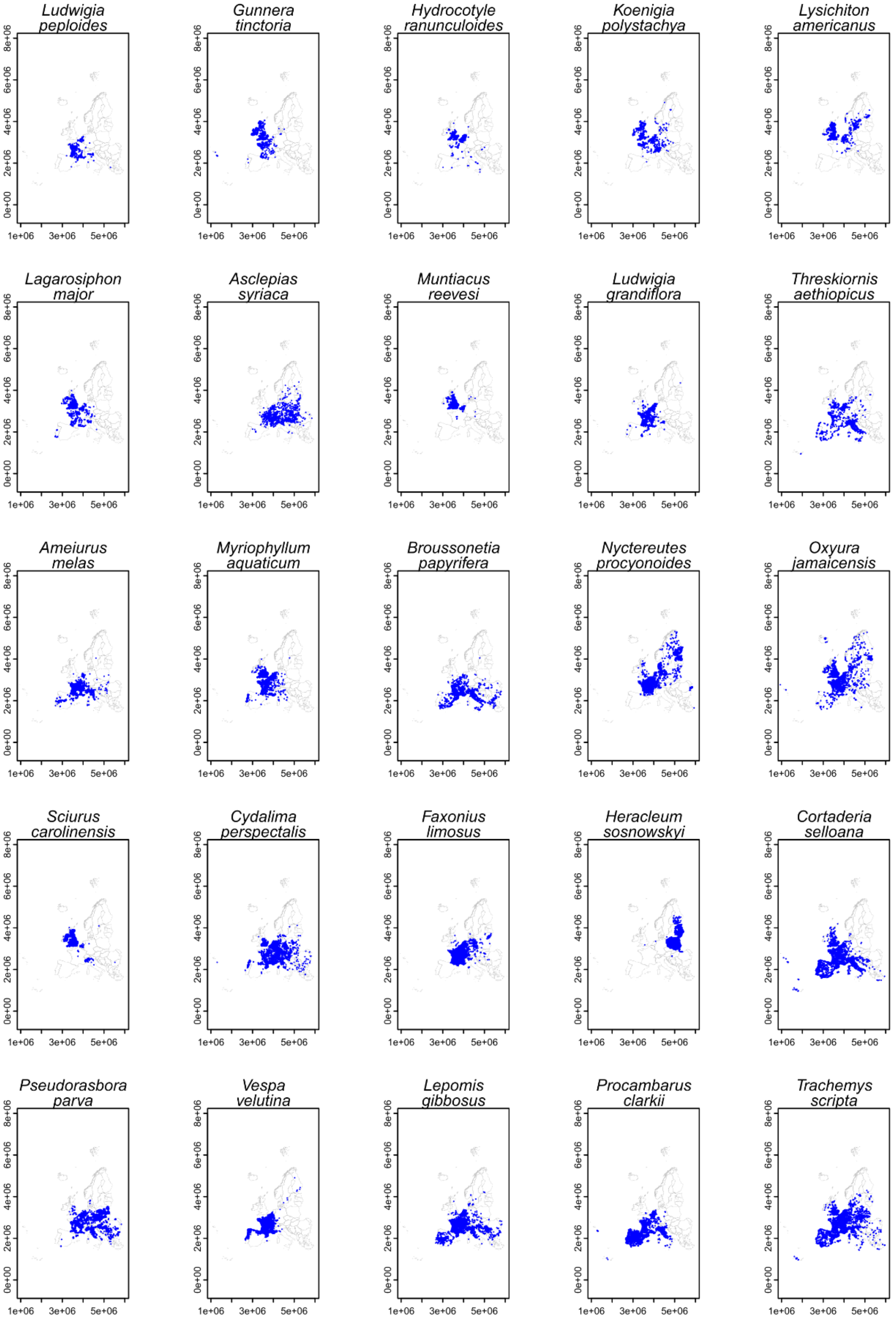
Ludwigia peploides
Gunnera tinctoria
Hydrocotyle ranunculoides
Koenigia polystachya
Lysichiton americanus
Lagarosiphon major
Asclepias syriaca
Muntiacus reevesi
Ludwigia grandiflora
Threskiornis aethiopicus
Ameiurus melas
Myriophyllum aquaticum
Broussonetia papyrifera
Nyctereutes procyonoides
Oxyura jamaicensis
Sciurus carolinensis
Cydalima perspectalis
Faxonius limosus
Heracleum sosnowskyi
Cortaderia selloana
Pseudorasbora parva
Vespa velutina
Lepomis gibbosus
Procambarus clarkii
Trachemys scripta
0e+00
2e+06
4e+06
6e+06
8e+06
1e+06
3e+06
5e+06

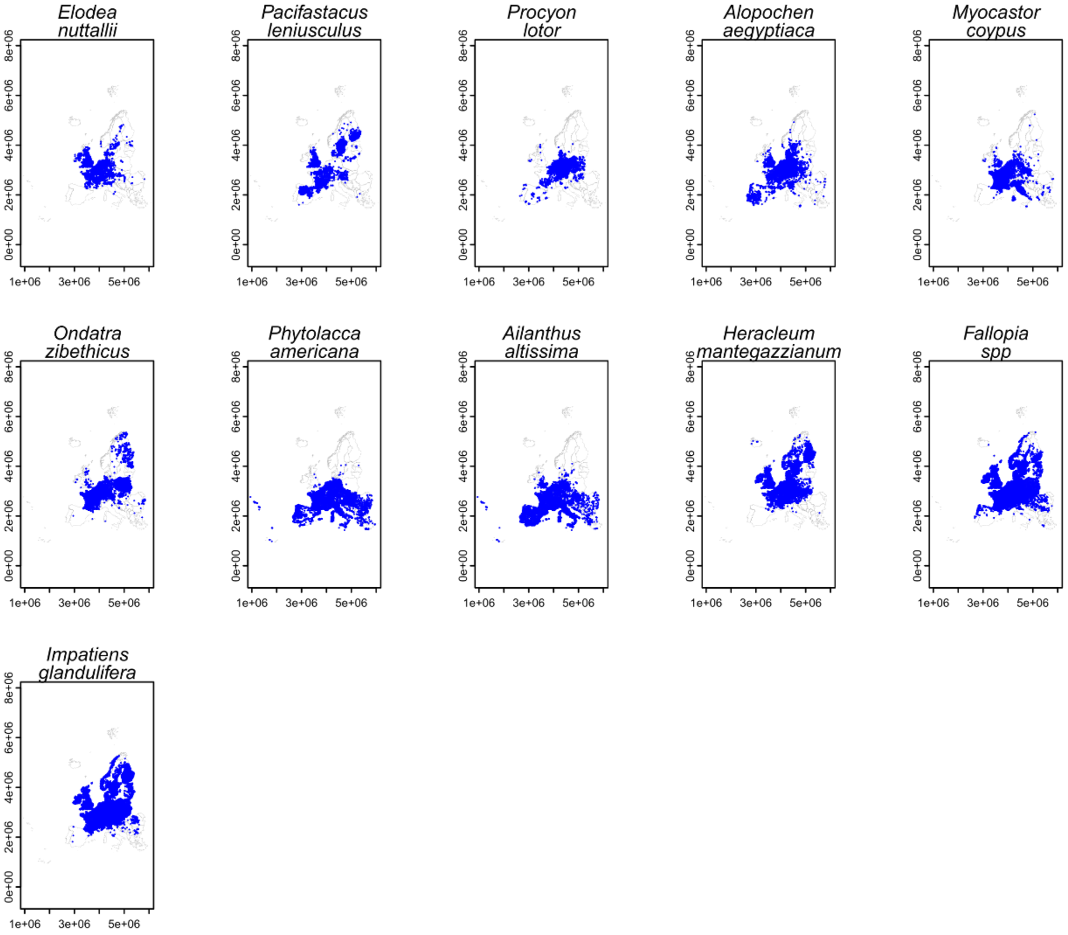
Elodea nuttallii
Pacifastacus leniusculus
Procyon lotor
Alopochen aegyptiaca
Myocastor coypus
Ondatra zibethicus
Phytolacca americana
Ailanthus altissima
Heracleum mantegazzianum
Fallopia spp
Impatiens glandulifera
0e+00
2e+06
4e+06
6e+06
8e+06
1e+06
3e+06
5e+06

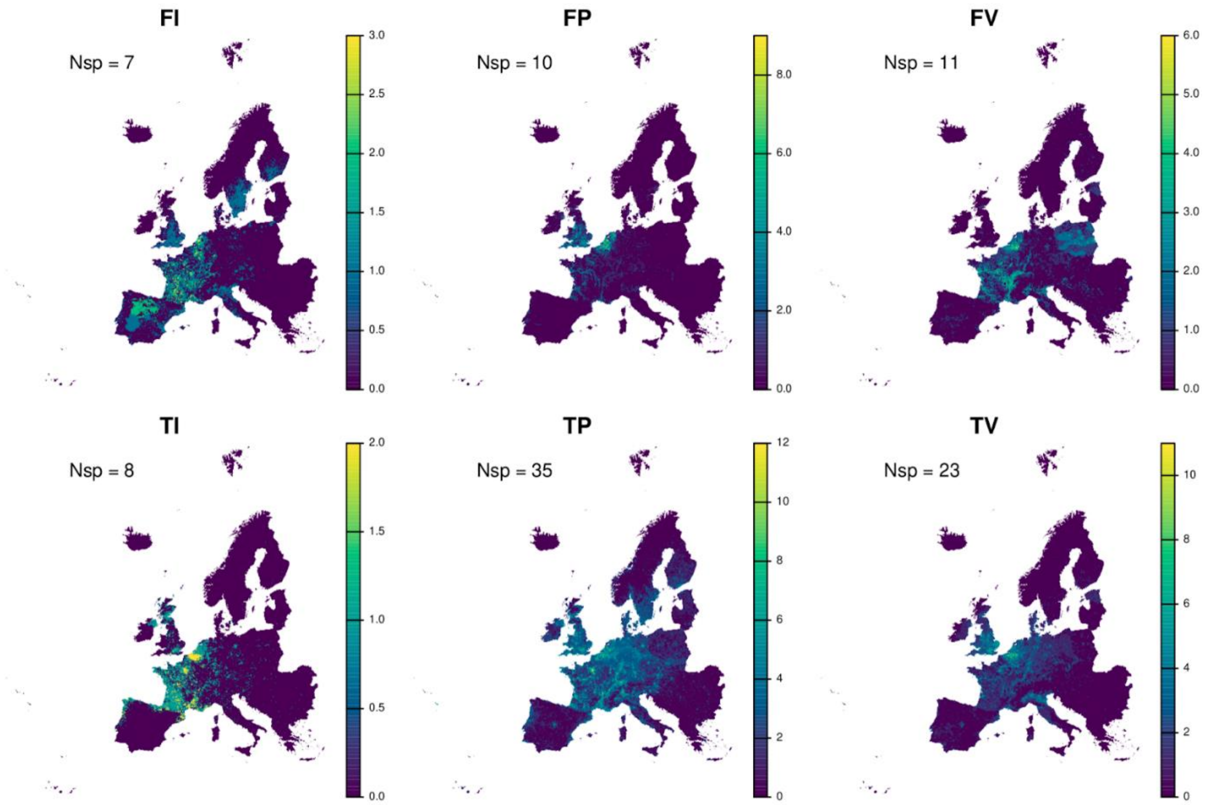

Supplementary Fig. 3. *Number of analysed species recorded on European 10x10 km grid cells by major taxonomic groups. FI= freshwater invertebrates, FP= freshwater plants, FV= freshwater vertebrates, TI= terrestrial invertebrates, TP= terrestrial plants, TV= terrestrial vertebrates. Nsp: total number of species in each group. Maps are in Lambert Azimuthal Equal-Area (LAEA) Europe projection. Sources of occurrences downloaded, ORCID codes can be consulted in Supplementary Data 2.*

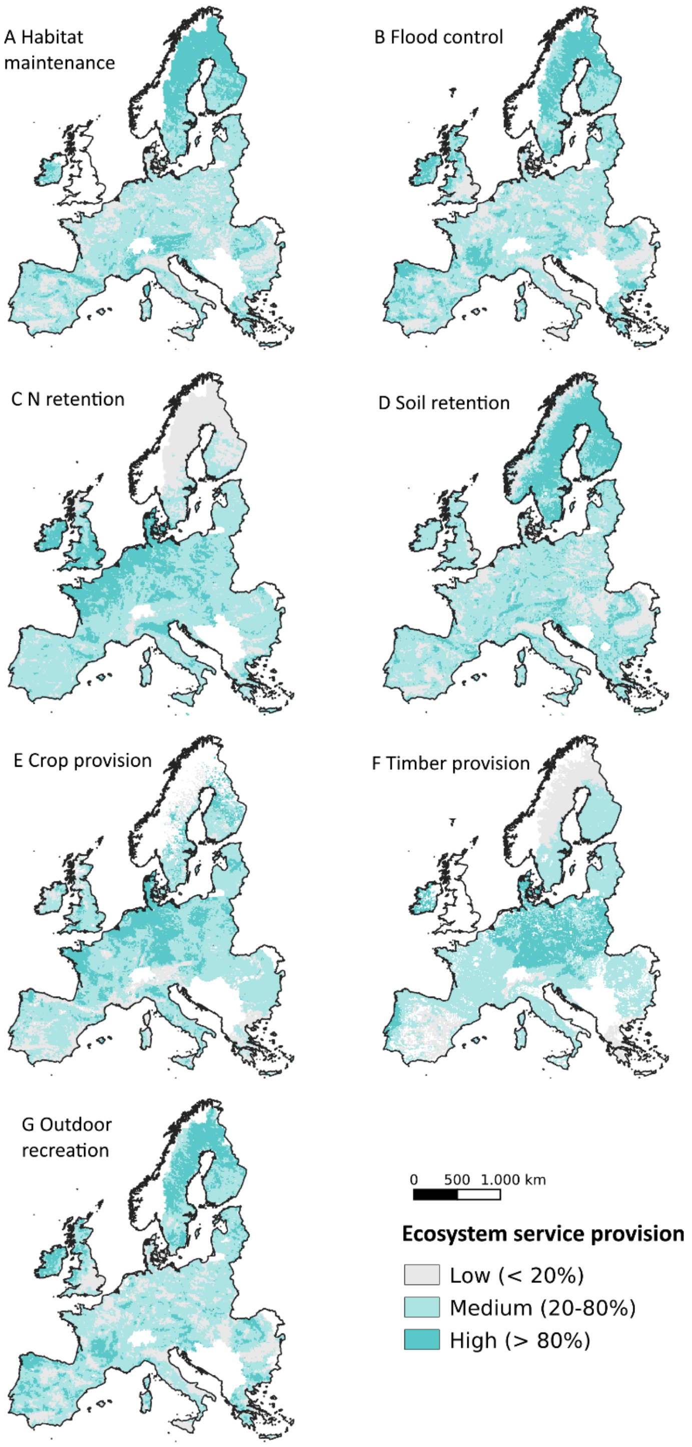

Supplementary Fig. 4. *Maps of the provision of ecosystem services reclassified into three quintiles: Low, Medium and High provisioning. A) Habitat maintenance. B) Flood control. C) Nitrogen retention. D) Soil retention. E) Crop provision. F) Timber provision. G) Outdoor recreation. Raw data on the provision of ecosystem services was obtained from the European Environment Agency, through the Joint Research Center (https://data.jrc.ec.europa.eu/dataset).*

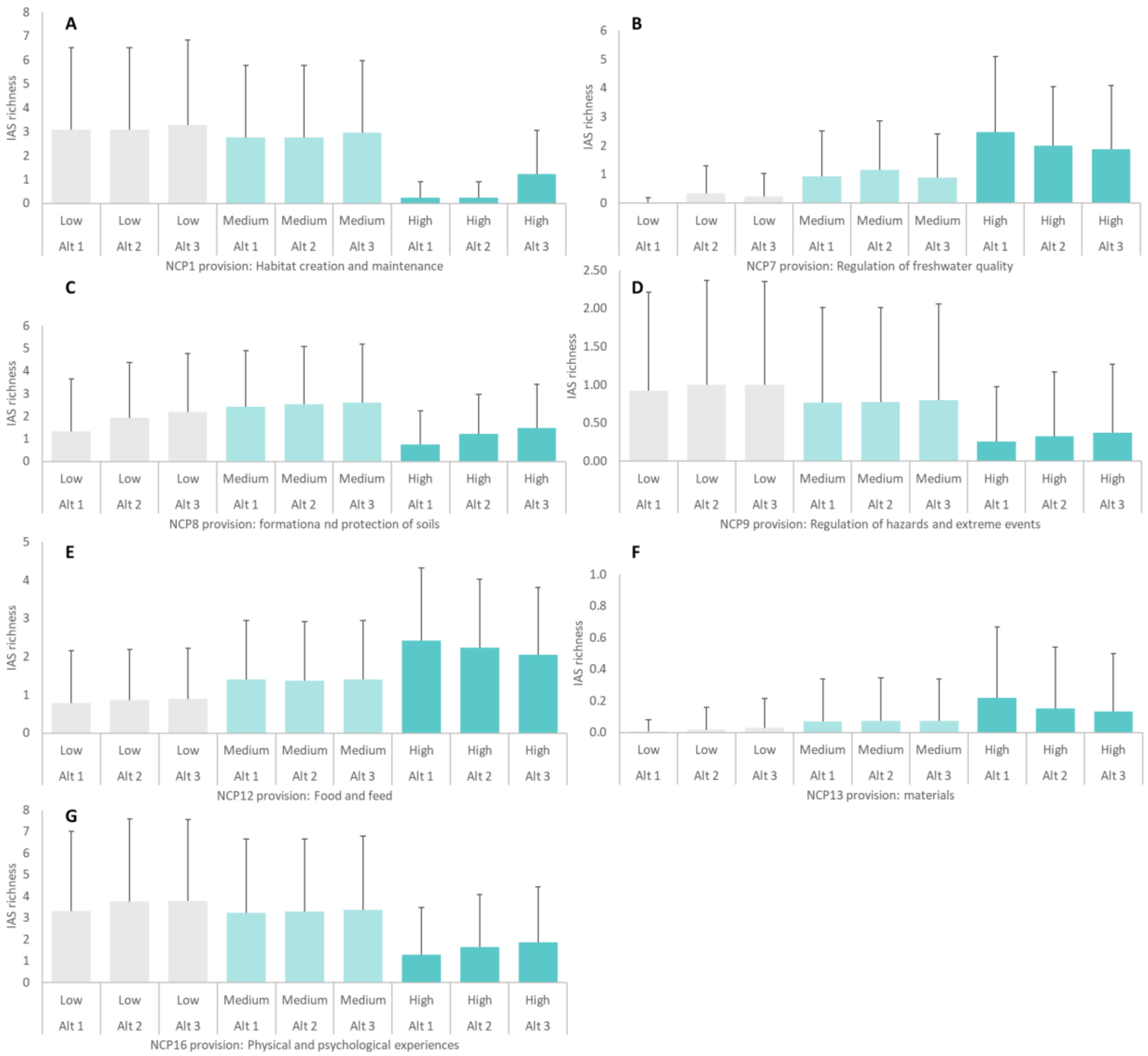

Supplementary Fig. 5. *Sensitivity analysis using three alternative thresholds to measure the current threat posed by invasive species on ecosystem service provision. A) Habitat maintenance. B) Nitrogen retention. C) Soil retention. D) Flood control. E) Crop provision. F) Timber provision. G) Outdoor recreation. Bars represent the mean+SD number of invasive species know to occur per pixel of 10x10 km in Europe. Alt 1/2/3 represent the three alternative thresholds to classify service provisioning into Low/Medium/High categories.*

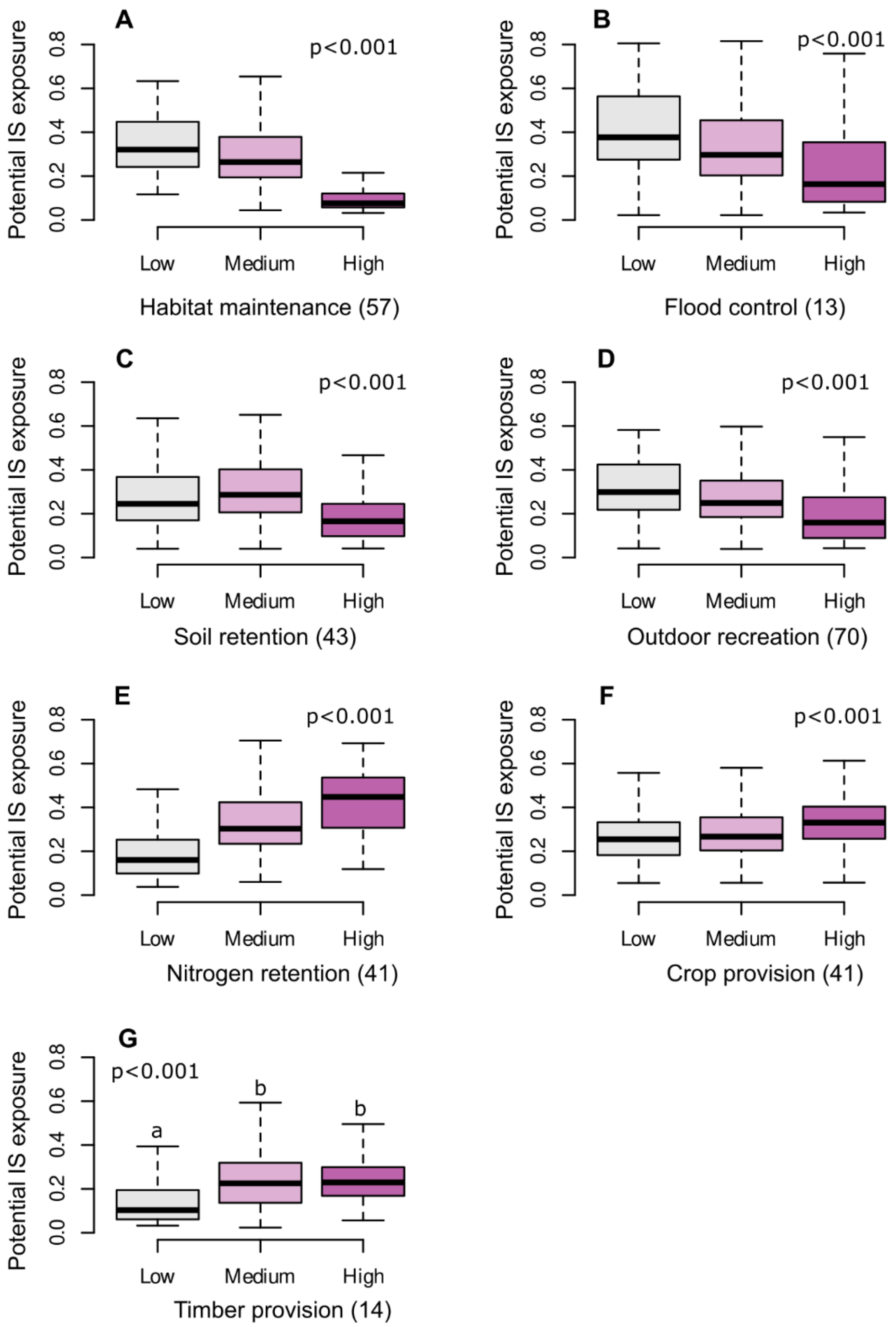

Supplementary Fig. 6 *Potential exposure of ecosystem services to invasive species. A) Habitat maintenance. B) Flood control. C) Soil retention. D) Outdoor recreation. E) Nitrogen retention. F) Crop provision. G) Timber provision. Potential exposure is measured as the number of invasive species per 10x10 km cell with high favourability, predicted to occur in areas delivering Low, Medium and High ecosystem services. Values are rescaled to 0-1 to account for the uneven number of invasive species that can affect each service individually, indicated in brackets. Box-plots represent the median (centre line), interquartile interval (box), and 1.5x interquartile range. P-values correspond to Welch one-way ANOVA. Pairwise Tukey HSD post-hoc comparisons were all highly significant except for timber provision (G), for which letters indicate significantly different groups.*

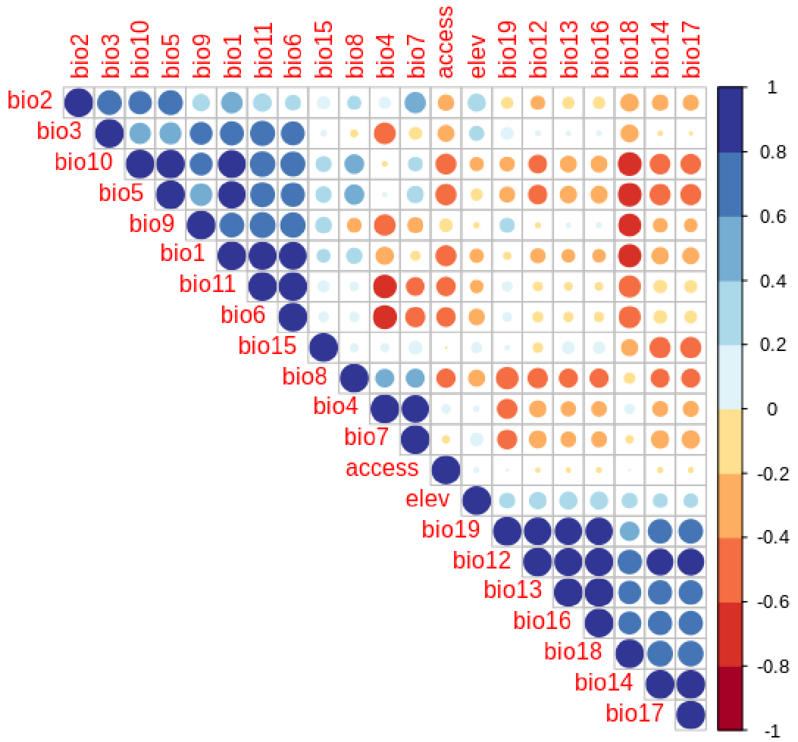

Supplementary Fig. 7. *Correlations among the 21 candidate variables. access= accessibility, elev= elevation. The colour scale at the right refers to Pearson's r coefficient. Darker colours represent stronger positive (red) or negative (bue) correlations. bio1= mean annual air temperature; bio2= mean diurnal air temperature range; bio3= isothermality; bio4= temperature seasonality; bio5= mean daily maximum air temperature of the warmest month; bio6= mean daily minimum air temperature of the coldest month; bio7= annual range of air temperature; bio8= mean daily mean air temperatures of the wettest quarter; bio9= mean daily mean air temperatures of the driest quarter; bio10= mean daily mean air temperatures of the warmest quarter; bio11= mean daily mean air temperatures of the coldest quarter; bio12= annual precipitation amount; bio13= precipitation amount of the wettest month; bio14= precipitation amount of the driest month; bio15= precipitation seasonality; bio16= mean monthly precipitation amount of the wettest quarter; bio17= mean monthly precipitation amount of the driest quarter; bio18= mean monthly precipitation amount of the warmest quarter; bio19= mean monthly precipitation amount of the coldest quarter.*

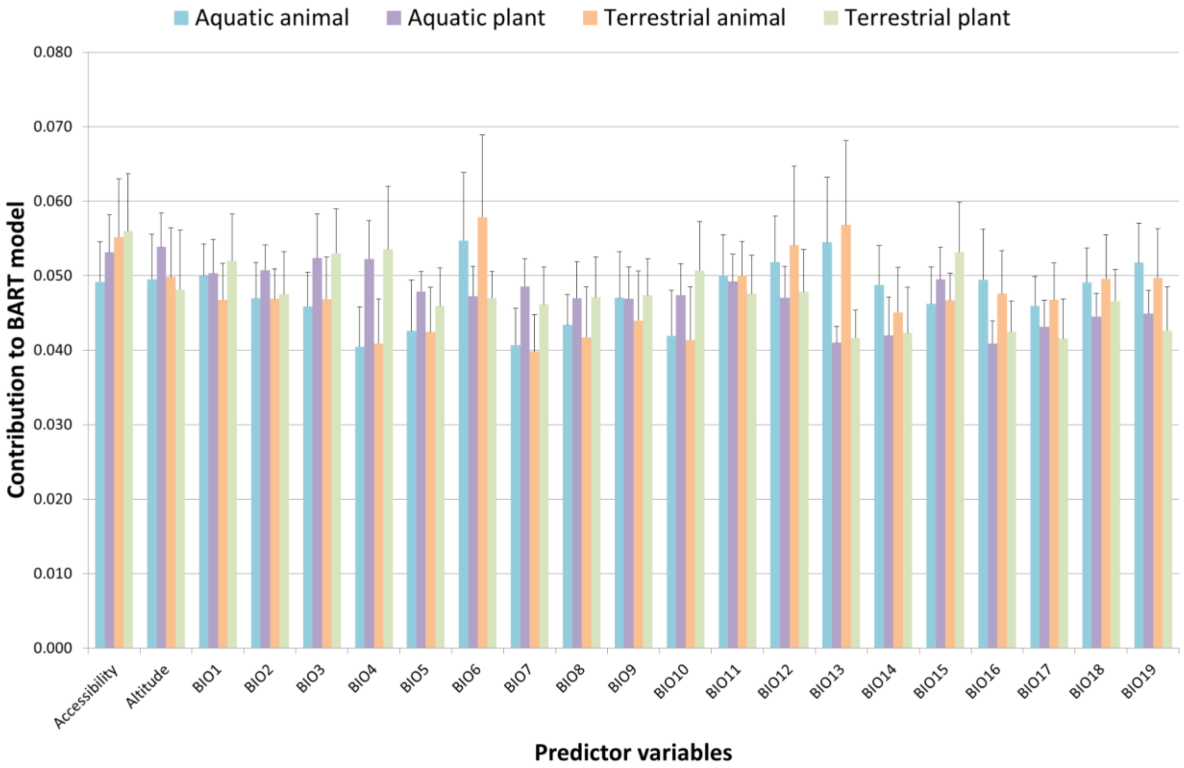

Supplementary Fig. 8. *Contribution of predictor variables to species distribution models. Values represent the mean and standard deviation across 32 terrestrial plants, 29 terrestrial animals, 20 aquatic animals and 13 aquatic plants.*

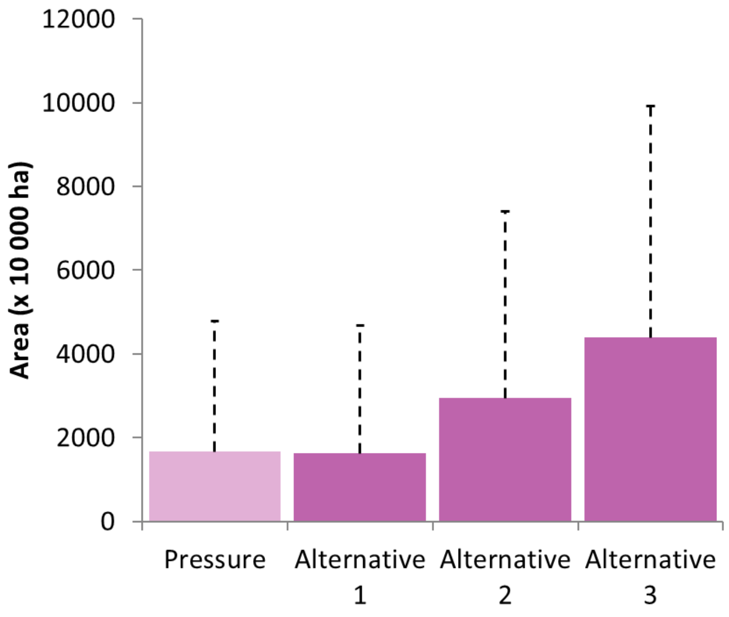

Supplementary Fig. 9. *Barplot showing the average (+standard deviation) area under current and potential threat by invasive species of concern in Europe, using three alternative threat thresholds: favourability $\geq$0.90, $\geq$0.80 and $\geq$0.70 respectively.*

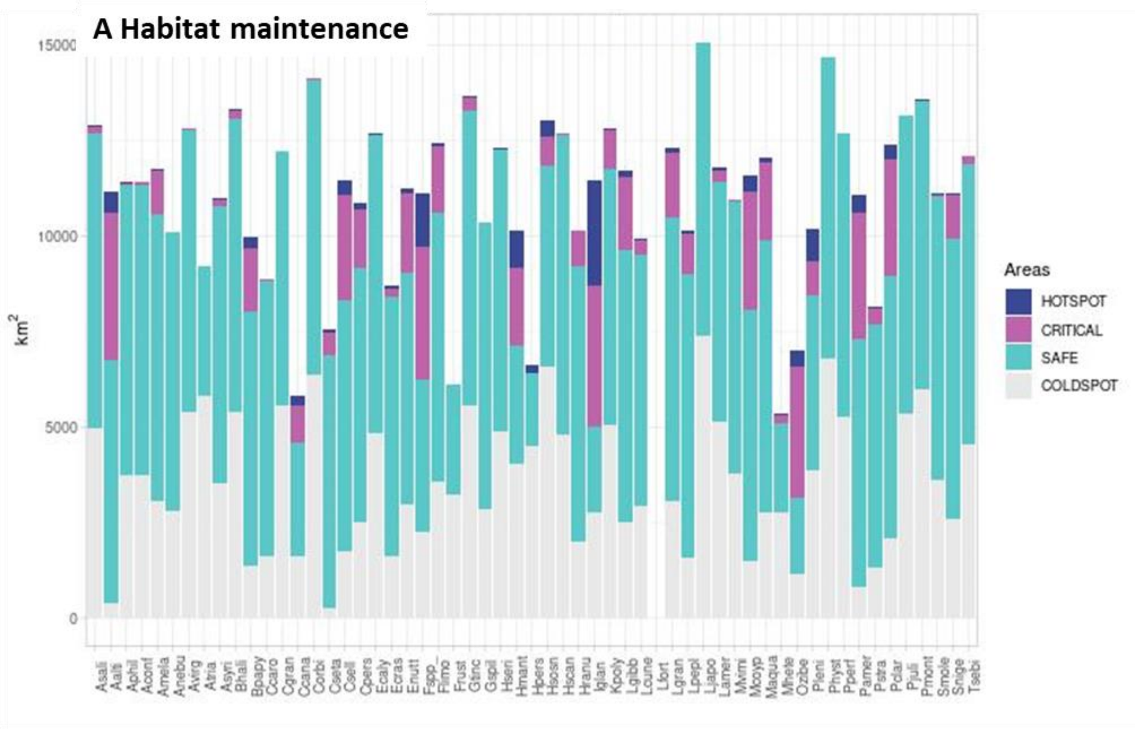
A Habitat maintenance
15000
10000
5000
0
km^2
Areas
HOTSPOT
CRITICAL
SAFE
COLDSPOT
Asali
Aalli
Aphil
Aconf
Amelia
Anebu
Avirg
Atria
Asyn
Bhali
Bpapy
Ccaro
Cgran
Ccana
Corbi
Cseta
Csell
Cpers
Ecaly
Ecras
Enutt
Fspp_
Fiimo
Frust
Gfinc
Gspil
Hseri
Hmant
Hpers
Hscsn
Hscan
Hranu
Iglan
Kpoly
Lgibb
Loune
Lfort
Lgran
Lpepl
Ljapo
Lamer
Mvimi
Mcoyp
Maqua
Mhete
Ozibe
Pleni
Physt
Pperl
Pamer
Pstra
Pclar
Pjuli
Pmont
Smole
Snige
Tsebi

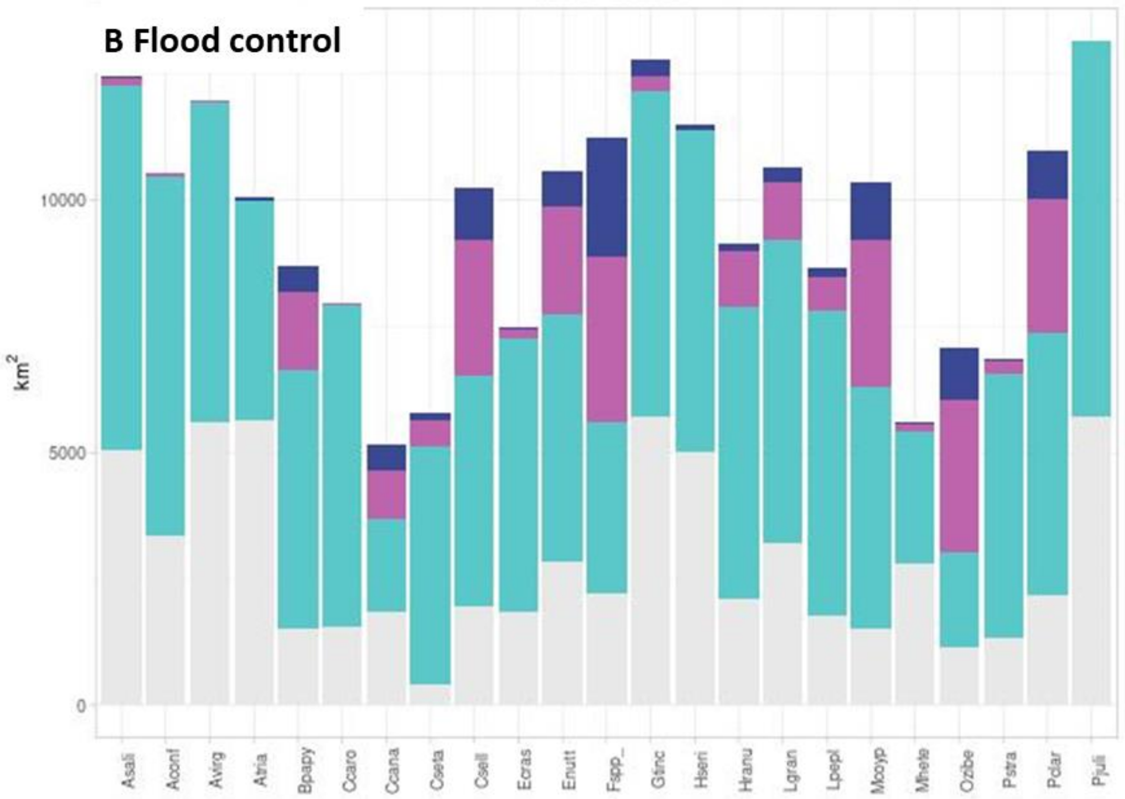
B Flood control
10000
5000
0
km^2
Asali
Aconf
Avirg
Atria
Bpapy
Ccaro
Ccana
Cseta
Csell
Ecras
Enutt
Fspp_
Gfinc
Hseri
Hranu
Lgran
Lpepl
Mcoyp
Mhete
Ozibe
Pstra
Pclar
Pjuli

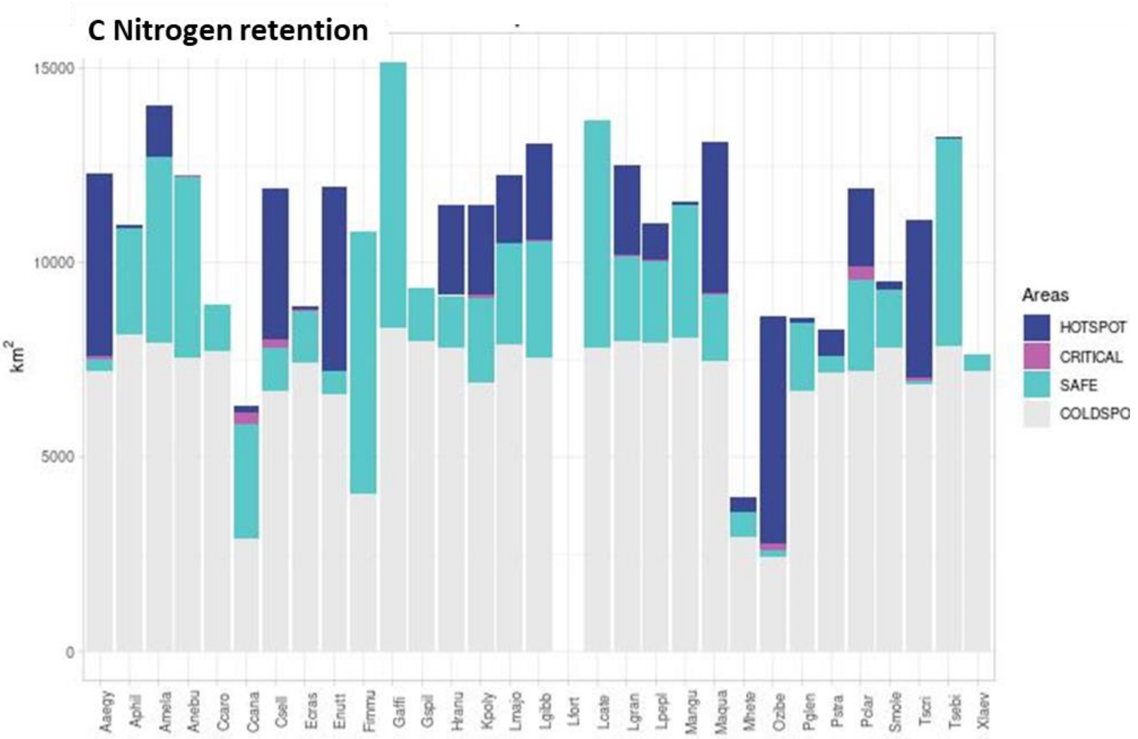

C Nitrogen retention
15000
10000
5000
0
km2
Areas
HOTSPOT
CRITICAL
SAFE
COLDSPO
Aaegy
Aphil
Amela
Anebu
Ccaro
Ccana
Csell
Ecras
Enutt
Fimmu
Gaffi
Gspil
Hranu
Kpoly
Lmajo
Lgibb
Lfort
Lcate
Lgran
Lpepl
Mangu
Maqua
Mhete
Ozibe
Pglen
Pstra
Pcolar
Smole
Tscri
Tsebi
Xlaev

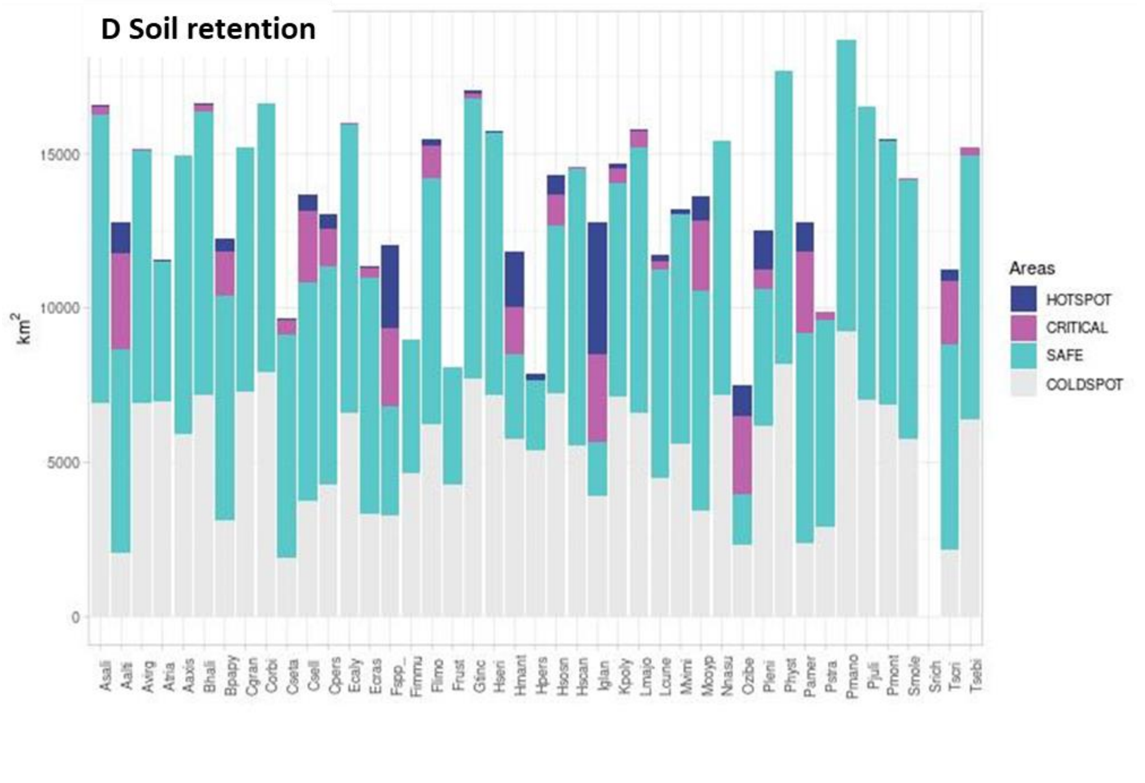

D Soil retention
15000
10000
5000
0
km2
Areas
HOTSPOT
CRITICAL
SAFE
COLDSPOT
Asali
Aalti
Avirg
Atria
Aaxis
Bhali
Bpapy
Cgran
Corbi
Cseta
Csell
Cpers
Ecaly
Ecras
Fspp_
Fimmu
Flimo
Frust
Gtinc
Hseri
Hmant
Hpers
Hsosn
Hscan
Iglan
Kpoly
Lmajo
Lcune
Mvimi
Mcoyp
Nnasu
Ozibe
Pleni
Physt
Pamer
Pstra
Pmano
Pjuli
Pmont
Smole
Srich
Tscri
Tsebi

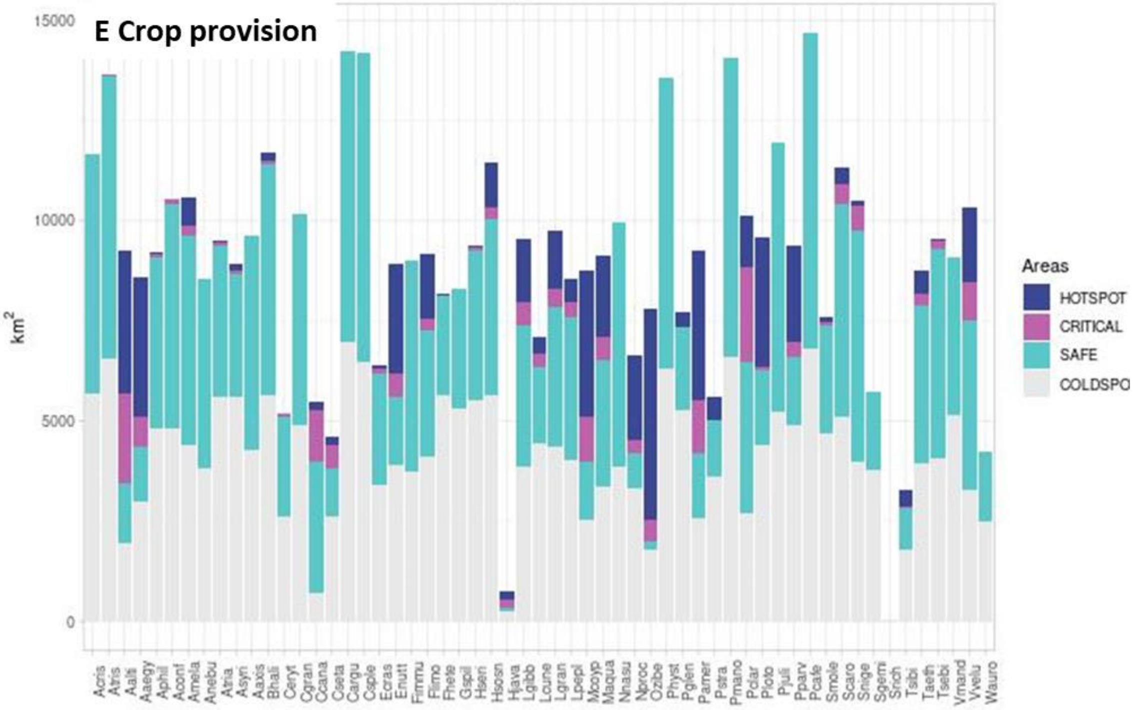
E Crop provision
15000
10000
5000
0
km^2
Areas
HOTSPOT
CRITICAL
SAFE
COLDSPO
Acris Atris Aalti Aaegy Aphil Aconf Amelia Anebu Atria Asyri Aaxis Bhali Ceryt Cgran Ccana Cseta Cargu Caple Ecras Enutt Fimmu Flimo Fhete Gspil Hseri Hsosn Hjava Lgibb Loune Lgran Lpepl Mooyp Maqua Nnasu Nproc Ozibe Physt Pglen Pamer Pstra Pmano Pclar Ploto Pjuli Pparv Pcafe Smole Scaro Snige Sgemi Srich Tsibi Taeth Tsebi Vmand Vvelu Wauro

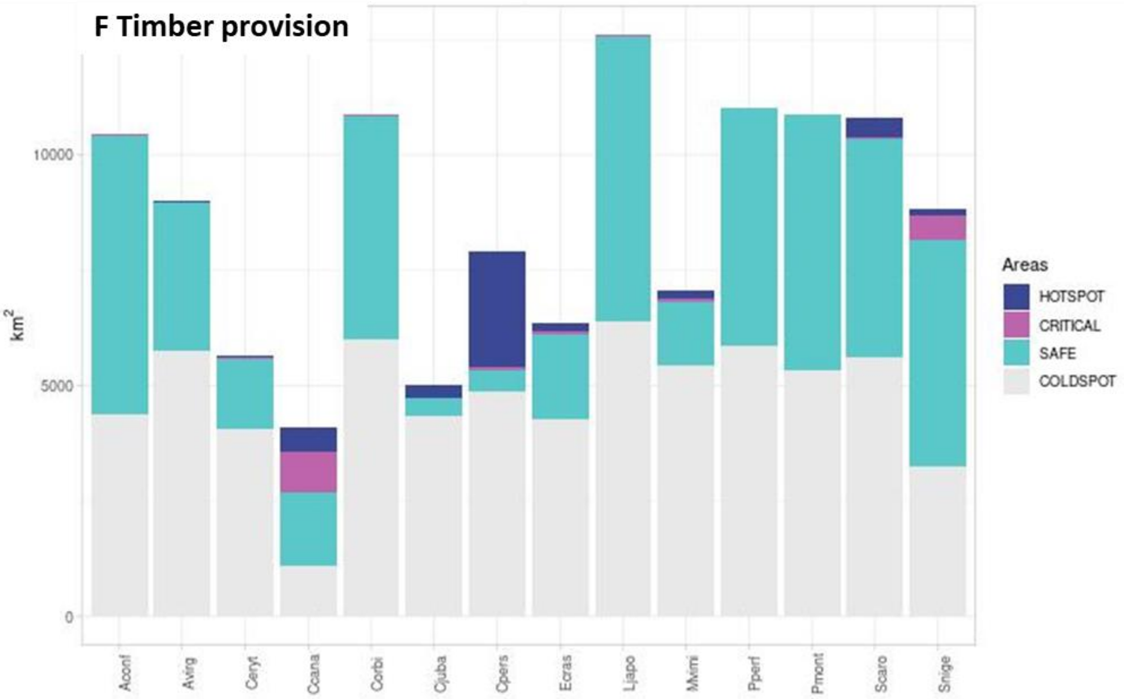
F Timber provision
10000
5000
0
km^2
Areas
HOTSPOT
CRITICAL
SAFE
COLDSPOT
Aconf Avirg Ceryt Ccana Corbi Cjuba Opers Ecras Ljapo Mvimi Pperl Pmont Scaro Snige

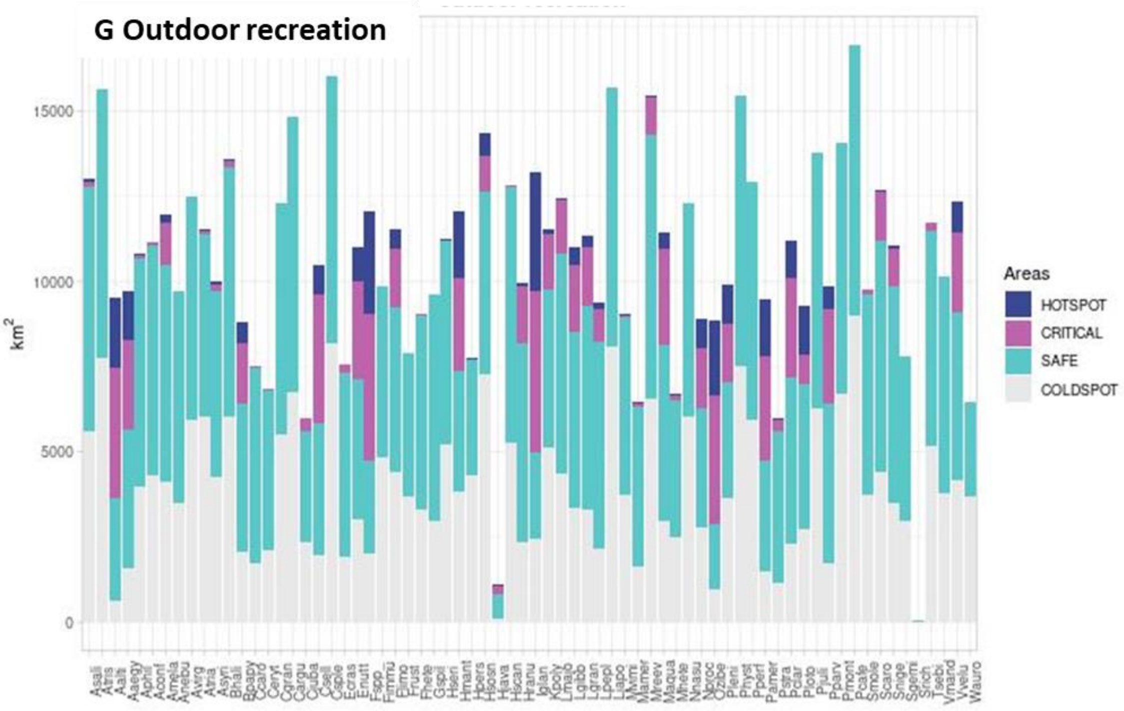

Supplementary Fig. 10. *Total areas of Europe that are classified into 4 impact categories combining the favourability for invasive species and ecosystem services provision. A) Habitat maintenance. B) Flood control. C) Nitrogen retention. D) Soil retention. E) Crop provision. F) Timber provision. G) Outdoor recreation. "Critical" represents sites where ecosystem service provision is low and the probability of invasion is high, "Hotspots" represents sites combining a high probability of invasion and a high delivery of ecosystem services. In the x-axis, ID of invasive species with reported evidence of negative impact upon the specific service. The full name and characteristics of invasive species can be consulted in Supplementary Table 1.*

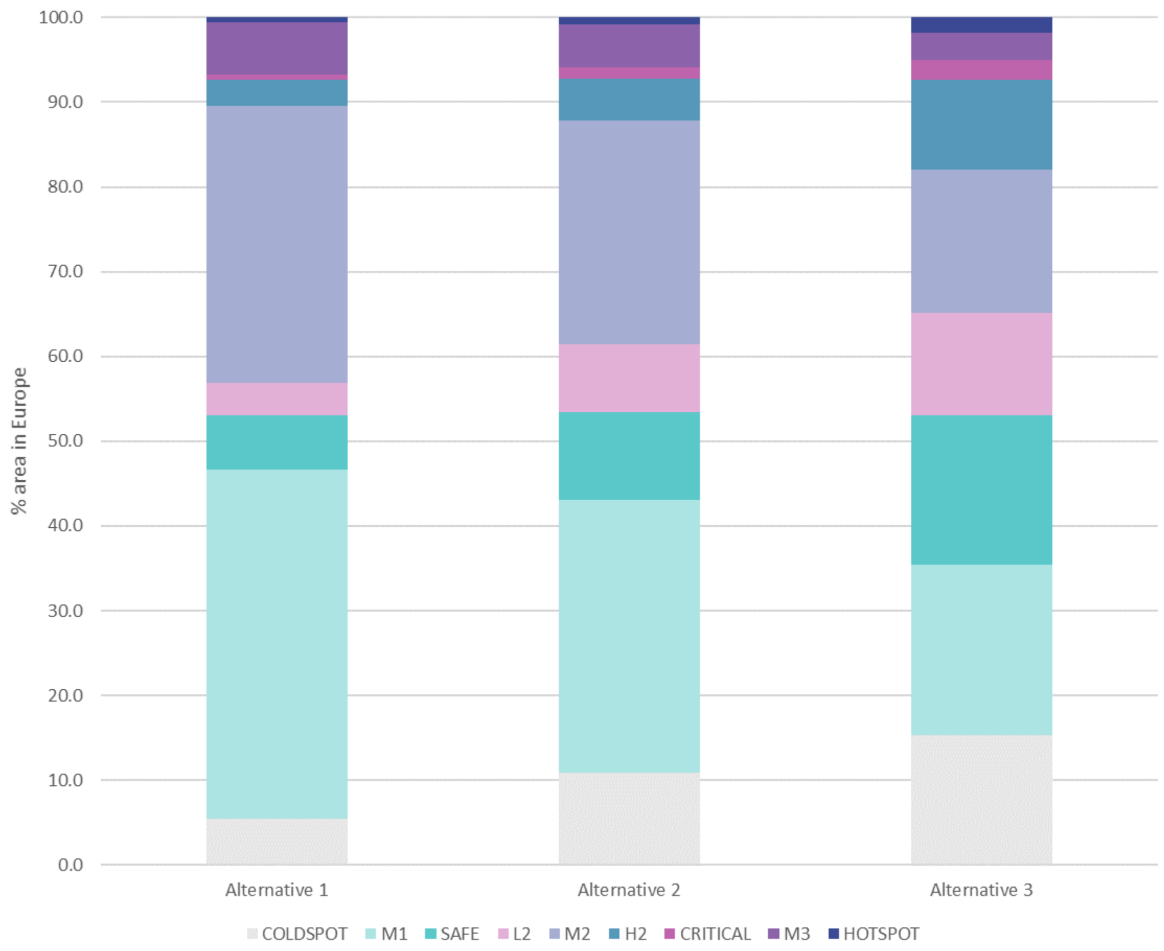

Supplementary Fig. 11. *Sensitivity analysis of the effects of alternative thresholds used to classify ecosystem services into Low-Medium-High delivering areas. Alternative 2 is used in the main manuscript.*

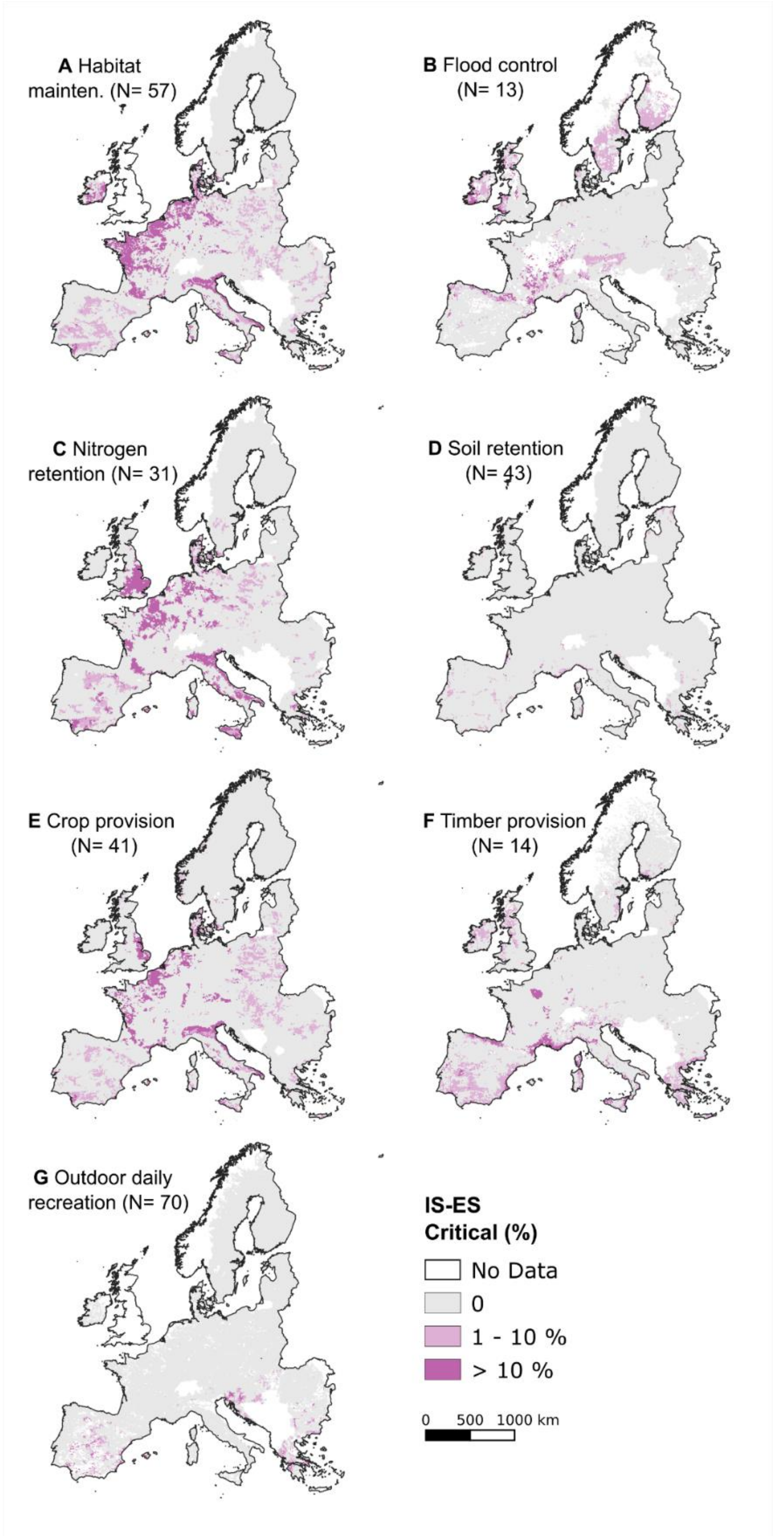

Supplementary Fig. 12. *Density of critical areas for invasive species potential impacts on ecosystem services (ES). A) Habitat maintenance. B) Flood control. C) Nitrogen retention. D)Soil retention. E) Crop provision. F) Timber provision. G) Outdoor daily recreation. Critical areas combine high favourability for invasion and low delivery of ES. Values represent the % of invasive species known to negatively affect each specific ES that are either present or likely to be present in the near future in low-ES provisioning areas. The total number of invasive species of European concern that are known to affect each ES is indicated in brackets. Raw data on the provision of ecosystem services was obtained from the European Environment Agency, through the Joint Research Center (https://data.jrc.ec.europa.eu/dataset).*

Supplementary Tables

Supplementary Table 1. *Ecosystem services investigated in this study, including their metadata. Resolution: the resolution of the original files, which is converted to 10x10 km for our analysis. Ref Year: reference year used for analyses. EU Area Covered: because of the UK exit, the number of EU countries covered by the different maps differed. The number of invasive species of European concern that have reported negative impacts on each ecosystem services can be consulted in Supplementary Table 1. Raw data on the provision of ecosystem services was obtained from the European Environment Agency, through the Joint Research Center (https://data.jrc.ec.europa.eu/dataset). References of each dataset, including accession links are indicated.*

DESCRIPTION OF THE PROXY	RESOLUTION	UNITS	REF YEAR	EU AREA COVERED	REFERENCE
**Habitat maintenance.** This map reflects suitable ecological conditions (or suitable habitats) required to support species populations, integrating information about the quality and quantity of different ecosystem types. The habitat suitability indicator is calculated by multiplying the ecological condition of European habitats, to account for habitat degradation, by the relative ecosystem extent per 10x10 km cell, to account for habitat loss.	10x10 km	unitless, from 0 to 100	2012	40.730 (x100 Km^2)	Zurbaran et al. [3]
**Nitrogen retention by natural ecosystems.** In large-scale assessments, nitrogen retention has been adopted as a proxy to quantify the service of water purification. The capacity of ecosystems to purify waters is mapped considering the nitrogen inputs, weather and hydrological conditions of 2012. The model considers basin retention (including crop uptake) and river and lake retention.	30 arcsec	tonne/ha	2012	38.898 (x100 Km^2)	Maes et al. [4]
**Soil retention.** Indicator that reflects the relationship between the capacity of ecosystems to avoid soil erosion and how much soil is retained by vegetation. Soil retention is a key regulatory service that refers to the ability of ecosystems to retain soil and minimise natural or induced erosion.	100 m^2	% of potential soil erosion that is mitigated by ecosystems. From 0 to 100.	2012	48.354 (x100 Km^2)	Maes et al. [4]
**Flood control.** Defined here as the regulation of water flow by ecosystems that mitigates or prevents potential damage to economic assets (i.e., infrastructure, agriculture) and human lives. This proxy measures the hectares of land benefiting from the flood control provided by ecosystems in a given year.	shapefile (polygons)	% service provisioning areas upstream. From 0 to 100.	2012	40.026 (x100 Km^2)	Vallecillo et al. [5]
**Ecosystem contribution to crop provision.** Ecosystem contribution to the growth of cultivated crops that can be harvested and used for the production of food, fibre and fuel. The quantification disentangles the yield generated by natural inputs (i.e. sunlight, wind, rainfall, evapotranspiration, soil) from what is generated by human inputs (i.e., planting, irrigation, chemical products). The map represents the percentage of the yield that can be attributed to the ecosystem contribution, and varies from 0, when yield is entirely derived from human inputs, to 1 when no human input is involved. At the EU level, ecosystem contribution to crop provision is about 21% of the total yield value. The rest is due to human inputs.	1 km^2	tonnes/km^3 attributable to the ecosystem contribution	2012	39.321 (x100 Km^2)	Vallecillo et al. [6]
**Timber provision.** Ecological contribution to the production of timber that can be harvested and used as raw material. This is calculated using the Net Annual Increment of Timber (NAI), which represents the gross annual increment minus losses due to natural tree mortality and the human contribution to timber provision.	1 km^2	m^3/year	2012	35.188 (x100 Km^2)	Vallecillo et al. [7]
**Outdoor Recreation.** Value of ecosystems with a	shapefile	% areas for daily	2012	40.026	Vallecillo et

high potential for daily use for recreation. Daily recreation includes a wide variety of practises including walking, jogging, running, bike riding, picnicking, observing flora and fauna, enjoying the surrounding beauty of the landscape, among a myriad of other possibilities. The map is based on the recreation opportunities spectrum (ROS), which represents a whole range of recreation opportunities categorised as a function of their level of provision and the distance to roads and residential areas.	(NUTS2)	recreation per admin unit. From 0 to 100.	(x100 Km^2)	al. [8]

Supplementary Table 2. *Analysis of Variance in Invasive Species richness (ISR) across low, medium and high ecosystem service (ES) provisioning areas. Results from Welch One-Way ANOVA with unequal variances across groups are shown. Analyses are two-sided and with no adjustments for multiple comparisons. The dependent variable is the observed richness of invasive species affecting each ES ($SR_{ES1\rightarrow7}$). The independent variable is the ES provision, a categorical variable with three levels: low, medium and high. Results from a post hoc Tukey HSD Test show pairwise significant differences. "***"= highly significant at p<0.0001; n.s.= not significant at p>0.05.*

FORMULA	ONE-WAY ANOVA TEST		TUKEY HSD TEST
	F (DF)	P-VALUE	
ISR_{ES1} ~ Habitat maintenance	8875.6 (2, 9834)	<0.001 (***)	Low-Medium ***<br>Low-High ***<br>Medium-High ***
ISR_{ES2} ~ Flood control	968.83 (2, 17315)	<0.001 (***)	Low-Medium ***<br>Low-High ***<br>Medium-High ***
ISR_{ES3} ~ N retention	1986 (2, 2709)	<0.001 (***)	Low-Medium ***<br>Low-High ***<br>Medium-High ***
ISR_{ES4} ~ Soil retention	1589.1 (2, 21318)	<0.001 (***)	Low-Medium ***<br>Low-High ***<br>Medium-High ***
ISR_{ES5} ~ Crop provision	1473.6 (2, 15550)	<0.001 (***)	Low-Medium ***<br>Low-High ***<br>Medium-High ***
ISR_{ES6} ~ Timber provision	500.73 (2, 15395)	<0.001 (***)	Low-Medium ***<br>Low-High ***<br>Medium-High n.s.
ISR_{ES7} ~ Outdoor recreation	1373 (2, 16923)	<0.001 (***)	Low-Medium ***<br>Low-High ***<br>Medium-High ***

Supplementary References

1. Wadoux, A. M.-C., Heuvelink, G. B., De Bruin, S. & Brus, D. J. Spatial cross-validation is not the right way to evaluate map accuracy. *Ecol. Model.* **457**, 109692 (2021).
2. Valavi, R., Elith, J., Lahoz-Monfort, J. & Guillera-Arroita, G. Package 'blockCV': Spatial and Environmental Blocking for K-Fold Cross-Validation. (2020).

3. Zurbaran, N., Vallecillo, S., La Notte, A. & Grammatikopoulou, I. INCA - Habitat and Species Maintenance. European Commission, Joint Research Centre (JRC) https://doi.org/PID: http://data.europa.eu/89h/fe1158c3-7801-4347-9e8b-fa634d1a0cd6 (2020).
4. Maes, J., Zurbaran Nuci, M., La Notte, A. & Grizzetti, B. INCA - Water Purification. European Commission, Joint Research Centre (JRC) https://doi.org/PID: http://data.europa.eu/89h/e32d63d1-dca8-4470-9775-62ad6cd79eca (2019).
5. Vallecillo, S., Kakoulaki, G., La Notte, A., Garcia Bendido, E. & Maes, J. INCA - Flood Control. European Commission, Joint Research Centre (JRC) https://doi.org/PID: http://data.europa.eu/89h/6b805df4-ee6e-4e42-a078-e0d6c72c40f7 (2019).
6. Vallecillo, S., La Notte, A., Garcia Bendido, E. & Maes, J. INCA - Crop Provision. European Commission, Joint Research Centre (JRC) https://doi.org/PID: http://data.europa.eu/89h/ecd792d1-61c3-478c-aa4a-587bad385805 (2020).
7. Vallecillo, S., La Notte, A., Garcia Bendido, E. & Maes, J. INCA - Timber Provision. European Commission, Joint Research Centre (JRC) https://doi.org/PID: http://data.europa.eu/89h/2583094a-0e1b-44c2-86cb-ad6a1ffe02fa (2020).
8. Vallecillo, S., La Notte, A., Zulian, G., Garcia Bendido, E. & Maes, J. INCA - Outdoor Recreation. European Commission, Joint Research Centre (JRC) https://doi.org/PID: http://data.europa.eu/89h/a6b63aee-da92-41fa-9812-cd769c7b32dc (2018).
