# Supplementary material for: Risks posed by invasive species to the provision of ecosystem services in Europe
Source: Nat Commun. 2024 Apr 10;15:2631. doi: 10.1038/s41467-024-46818-3 (PMC11006939; doi:10.1038/s41467-024-46818-3)
Supplement: Supplementary file 3 — Description of Additional Supplementary Files [file 41467_2024_46818_MOESM3_ESM.pdf]

Description of Additional Supplementary Files

File name: Supplementary Data 1

Description: Extended List of 94 Invasive Species of European Concern investigated in this study.

File name: Supplementary Data 2

Description: Sources of Invasive Species occurrence and total number of records used for modelling the potential distribution of 94 invasive species of European concern.

File name: Supplementary Data 3

Description: Results of model cross-validation for the 94 invasive species investigated. AUC= Area Under the receiver operating characteristic Curve. MCS= Millers calibration slope.

File name: Supplementary Data 4

Description: Potential impact of invasive species on the provision of ecosystem services. Data indicate the area of Europe classified into 9 categories of risk (Fig. 4A), from Safe to Hotspot. All individual combinations between 94 invasive species and 7 services that may be potentially affected are shown.
