# Supplementary material for: Risks posed by invasive species to the provision of ecosystem services in Europe
Source: Nat Commun. 2024 Apr 10;15:2631. doi: 10.1038/s41467-024-46818-3 (PMC11006939; doi:10.1038/s41467-024-46818-3)
Supplement: Supplementary file 8 — Reporting Summary [file 41467_2024_46818_MOESM8_ESM.pdf]

nature portfolio

Corresponding author(s): Belinda Gallardo

Last updated by author(s): Feb 26, 2024

Reporting Summary

Nature Portfolio wishes to improve the reproducibility of the work that we publish. This form provides structure for consistency and transparency in reporting. For further information on Nature Portfolio policies, see our Editorial Policies and the Editorial Policy Checklist.

Statistics

For all statistical analyses, confirm that the following items are present in the figure legend, table legend, main text, or Methods section.

n/a	Confirmed	
☐	☒	The exact sample size (*n*) for each experimental group/condition, given as a discrete number and unit of measurement
☒	☐	A statement on whether measurements were taken from distinct samples or whether the same sample was measured repeatedly
☐	☒	The statistical test(s) used AND whether they are one- or two-sided<br>*Only common tests should be described solely by name; describe more complex techniques in the Methods section.*
☐	☒	A description of all covariates tested
☐	☒	A description of any assumptions or corrections, such as tests of normality and adjustment for multiple comparisons
☐	☒	A full description of the statistical parameters including central tendency (e.g. means) or other basic estimates (e.g. regression coefficient) AND variation (e.g. standard deviation) or associated estimates of uncertainty (e.g. confidence intervals)
☐	☒	For null hypothesis testing, the test statistic (e.g. *F*, *t*, *r*) with confidence intervals, effect sizes, degrees of freedom and *P* value noted<br>*Give P values as exact values whenever suitable.*
☒	☐	For Bayesian analysis, information on the choice of priors and Markov chain Monte Carlo settings
☒	☐	For hierarchical and complex designs, identification of the appropriate level for tests and full reporting of outcomes
☐	☒	Estimates of effect sizes (e.g. Cohen's *d*, Pearson's *r*), indicating how they were calculated

Our web collection on statistics for biologists contains articles on many of the points above.

Software and code

Policy information about availability of computer code

Data collection	Data was downloaded from webpages indicated in the manuscript, no software was used for data collection.
Data analysis	Data was analyzed with the statistical program R using its interface "R Studio".<br>The version for R packages used is indicated here and in the text: 'blockCV' R package v 2.1.1 ; 'gam' R package v 1.22-2; 'gbm' R package v 2.1.9; 'spatialBlock' v 2.1.4; 'scrubr' R package v 0.3.2; 'fuzzySim' R package v 3.8; 'terra' R package v 1.5.21; 'exactextractr' v 0.6.1; 'dbarts' R package 0.9-21; R package 'modEvA' v 3.0<br>The R scripts used in this study are provided in a Figshare repository under accession code https://figshare.com/s/f5bd8287c1e391780879.

For manuscripts utilizing custom algorithms or software that are central to the research but not yet described in published literature, software must be made available to editors and reviewers. We strongly encourage code deposition in a community repository (e.g. GitHub). See the Nature Portfolio guidelines for submitting code & software for further information.

Data

Policy information about availability of data

All manuscripts must include a data availability statement. This statement should provide the following information, where applicable:

- Accession codes, unique identifiers, or web links for publicly available datasets
- A description of any restrictions on data availability
- For clinical datasets or third party data, please ensure that the statement adheres to our policy

The intermediate data files generated in this study are provided as Datasets in a Figshare repository under accession code https://figshare.com/s/f8b4cf965b71ea5b1577.

Research involving human participants, their data, or biological material

Policy information about studies with human participants or human data. See also policy information about sex, gender (identity/presentation), and sexual orientation and race, ethnicity and racism.

Reporting on sex and gender	n/a
Reporting on race, ethnicity, or other socially relevant groupings	n/a
Population characteristics	n/a
Recruitment	n/a
Ethics oversight	n/a

Note that full information on the approval of the study protocol must also be provided in the manuscript.

Field-specific reporting

Please select the one below that is the best fit for your research. If you are not sure, read the appropriate sections before making your selection.

☐ Life sciences ☐ Behavioural & social sciences ☒ Ecological, evolutionary & environmental sciences

For a reference copy of the document with all sections, see nature.com/documents/nr-reporting-summary-flat.pdf

Ecological, evolutionary & environmental sciences study design

All studies must disclose on these points even when the disclosure is negative.

Study description	To investigate the risks posed by invasive species to the provision of ecosystem services at continental (Europe) scale. We retrieved data on:<br>1) the global distribution of 94 invasive species, the number of occurrences used for each species is indicated in Suppl. Data 2.<br>2) the provision of 7 ecosystem services in Europe. Maps were rasterized to a 10x10 km resolution. The surface of Europe covered by each map is indicated in Suppl. Table 1.<br>3) Study design. To investigate differences in the richness of invasive species (IS) among areas displaying Low/Medium/High provision of ecosystems services, we used Welch One-Way ANOVA. Independent variable: the provision of ecosystem services, Dependent variable: IS. Sample size of each analysis indicated in Fig. 1 and Suppl. Table2. The study is not nested or hierarchical.
Research sample	Our analysis focused on the 94 non-marine organisms that have undergone full risk assessment and have been identified as management priorities at the European scale (last accessed August 2022, https://circabc.europa.eu/). This includes 33 terrestrial plants, 31 terrestrial animals, 18 freshwater animals and 12 freshwater plants.<br>We obtained worldwide occurrence data for the 94 invasive species from the Global Biodiversity Information Facility (GBIF, https://www.gbif.org; see Supplementary Data 2 for DOIs).<br>We included additional occurrences from: 1) the European Alien Species Information Network (EASIN; https://easin.jrc.ec.europa.eu/easin), 2) several papers compiling invasive occurrence records from additional sources (Supplementary Data 2).
Sampling strategy	In total, for the 94 invasive species investigated, we gathered >9 million occurrence records that after cleaning and gridding (i.e. selection of only one occurrence record per pixel of 10x10 km) resulted in 310,556 data points (see Supplementary Data 2 for a breakdown by species).<br>Species showing less than 20 occurrence points (Solenopsis japonica) were removed from further analysis.<br>Pseudo-absences were generated by randomly selecting separate 10-arc-minute pixels, in the same number of each species' presences, outside the presence pixels but within a 200-km spatial buffer around them. This aims to restrict the choice of pseudo-absences to potentially reachable areas in regions where the species has been surveyed, thus conferring pseudo-absences the same

	survey bias as presences, which is key to improve the transferability of models. This spatial selection was done using the 'terra' R package v 1.5.21.
Data collection	Data was collected by Belinda Gallardo and Marcia Barbosa.
Timing and spatial scale	Data on species occurrence is not restricted by time or space. It therefore includes all available historical records until 2021-2022 and is collected at the global scale. Specific dates of GBIF download (in 2021 or 2022 depending on the species) can be consulted in Suppl. Data 2.<br>Data on ecosystem services refers to the year 2012 and covers the European Union (+ UK for some services). Data coverage of each map is indicated in Suppl. Table 1.
Data exclusions	No data were excluded from analyses.
Reproducibility	Sensitivity analyses were perfomed to look at the robustness of conclusions when using alternative thresholds to classify variables into low/medium/high. To ensure reproducibility, all R codes are available through a Figshare repository.
Randomization	Randomization was used in species distribution models to: 1) calcuate the relative importance of predictors in the model, 2) spatial-block cross-validation.
Blinding	This study does not inclue any sensitive information requiring blinding.

Did the study involve field work? ☐ Yes ☒ No

Reporting for specific materials, systems and methods

We require information from authors about some types of materials, experimental systems and methods used in many studies. Here, indicate whether each material, system or method listed is relevant to your study. If you are not sure if a list item applies to your research, read the appropriate section before selecting a response.

Materials & experimental systems

n/a	Involved in the study
☒	☐ Antibodies
☒	☐ Eukaryotic cell lines
☒	☐ Palaeontology and archaeology
☒	☐ Animals and other organisms
☒	☐ Clinical data
☒	☐ Dual use research of concern
☒	☐ Plants

Methods

n/a	Involved in the study
☒	☐ ChIP-seq
☒	☐ Flow cytometry
☒	☐ MRI-based neuroimaging

Plants

Seed stocks	n/a
Novel plant genotypes	n/a
Authentication	n/a
